# 全血置换治疗霍奇金淋巴瘤合并危重自身免疫性溶血性贫血患者1例报告并文献复习

**DOI:** 10.3760/cma.j.cn121090-20260428-00219

**Published:** 2026-08

**Authors:** 冰清 李, 妍 谢, 子溢 刘, 佩淇 梁, 婷 徐, 鹏 王, 李 李, 菊萍 翟, 敏 蒋, 建红 付, 德沛 吴

**Affiliations:** 1 苏州大学附属第一医院血液科重症监护病房，血液系统疾病国家临床医学研究中心，江苏省血液研究所，苏州大学附属第一医院，江苏省血液病重点实验室，苏州 215006 Hematological intensive care unit, Department of Hematology, National Clinical Research Center for Hematologic Diseases, Jiangsu Institute of Hematology, Jiangsu Key Laboratory of Hematologic Diseases, The First Affiliated Hospital of Soochow University, Suzhou 215006, China; 2 苏州大学附属第一医院输血科，苏州 215006 Department of Blood Transfusion, the First Affiliated Hospital of Soochow University, Suzhou 215006, China

## Abstract

本文报道1例霍奇金淋巴瘤（HL）合并难治性危重自身免疫性溶血性贫血（AIHA）的救治过程，并探讨全血置换（WBE）在常规治疗及血浆置换失败后的应用价值。患者为52岁女性，有HL病史，曾接受抗PD-1单抗治疗，因进行性乏力、意识异常入院，血红蛋白最低为24 g/L，直接抗人球蛋白试验抗IgG及抗C3d均阳性（1∶256），伴有神志障碍及休克，确诊为危重AIHA。大剂量糖皮质激素、静脉免疫球蛋白、利妥昔单抗、依库珠单抗及血浆置换等多线治疗均无效，患者反复出现血压下降，经多学科会诊后实施WBE。患者术后24 h内尿色由浓茶色转为淡黄色，未再出现酱油色尿；术后第5天血红蛋白升至52 g/L，术后第10天升至63 g/L；乳酸脱氢酶由峰值2 304 U/L降至481 U/L，总胆红素降至20.4 µmol/L。患者循环稳定，于术后第16天转出血液重症监护病房。对于常规免疫抑制及血浆置换治疗无效的重症AIHA，WBE可快速清除致敏红细胞及补体成分，是有效的挽救性治疗手段。

## Abstract (en)

This article reports the management of a patient with Hodgkin lymphoma（HL）complicated by refractory critical autoimmune hemolytic anemia（AIHA）and explores the value of whole blood exchange（WBE）after failure of conventional therapy and plasma exchange. The patient was a 52-year-old woman with a history of classical HL treated with anti-PD-1 therapy. She was admitted due to progressive fatigue and altered mental status, with a nadir hemoglobin level of 24 g/L and a positive direct antiglobulin test for both anti‑immunoglobulin G and anti-C3d（1:256）. She also presented with impaired consciousness and shock, leading to a diagnosis of critical AIHA. The patient failed to respond to multiple lines of treatment, including high-dose corticosteroids, intravenous immunoglobulin, rituximab, eculizumab, and plasma exchange, and experienced recurrent hypotension. After multidisciplinary consultation, WBE was performed. Within 24 hours after the procedure, her urine color turned from dark tea-colored to light yellow, with no recurrence of dark urine. The hemoglobin level increased to 52 g/L on day 5 and to 63 g/L on day 10 post‑procedure. The lactate dehydrogenase level decreased from a peak level of 2 304 U/L to 481 U/L, and the total bilirubin level dropped to 20.4 µmol/L. The patient's hemodynamic status stabilized, and she was transferred out of the hematological intensive care unit on day 16. The present case demonstrates that for severe AIHA refractory to conventional immunosuppression and plasma exchange, WBE can rapidly remove sensitized erythrocytes and complement components, serving as an effective rescue therapy.

自身免疫性溶血性贫血（autoimmune hemolytic anemia，AIHA）是一类由机体免疫系统功能紊乱导致自身抗体介导红细胞破坏的获得性溶血性疾病。AIHA可独立发生，也可继发于感染、恶性肿瘤及自身免疫性疾病等[1]。严重的AIHA可迅速进展，甚至危及生命，临床表现为HGB急剧下降、酱油色尿、意识障碍、循环不稳定以及多器官功能衰竭。AIHA的常规治疗包括糖皮质激素、静脉丙种球蛋白、利妥昔单抗及补体抑制剂等[2]，但对于危重症患者，单纯血浆置换难以有效清除已结合于红细胞表面的抗体及补体成分。全血置换（whole blood exchange，WBE）作为一种融合血浆置换与红细胞清除的新型治疗技术，可同时去除循环中的致病抗体及致敏红细胞，为难治性危重AIHA提供了新的治疗选择[3]。

本文报道1例霍奇金淋巴瘤（Hodgkin lymphoma，HL）合并AIHA的危重患者，在常规治疗及血浆置换失败后，通过WBE成功救治的过程，旨在为类似患者的临床救治提供参考，并探讨WBE在危重AIHA中的应用价值。

## 病例资料

患者，女性，52岁，因“确诊HL 1年余，乏力半月余，神志异常1 d”于2026年1月27日入我院血液科重症监护病房（hematology intensive care unit，HCU）。患者于2024年3月诊断为经典型HL（混合细胞型，Ⅳ期B组），既往接受过BV-AVD方案（维布妥昔单抗50 mg第0天，脂质体多柔比星20 mg第1天，达卡巴嗪0.5 g第1天，长春地辛4 mg第1天）及抗PD-1单抗（替雷利珠单抗）联合AVD方案治疗，末次化疗时间为2025年12月20日。治疗期间曾出现严重肺炎、高热、皮疹等不良反应，考虑为抗PD-1单抗相关不良反应，经糖皮质激素对症治疗后好转。2026年1月初，患者出现进行性乏力、恶心呕吐。1月26日于我院急诊就诊时出现意识障碍及休克，血常规：WBC 4.07×10^9^/L、HGB 42 g/L、PLT 130×10^9^/L；生化：总胆红素42.3 µmol/L、LDH 1 603 U/L。次日凌晨患者出现高热、酱油色尿、神志模糊，遂转入HCU。

体格检查：患者体温39.1°C，脉率98次/min，呼吸14次/min，血压104/62 mmHg（1 mmHg＝0.133 kPa）；神志清、对答切题，贫血貌，巩膜黄染。辅助检查：血常规：WBC 2.26×10^9^/L、HGB 39 g/L、PLT 66×10^9^/L；网织红细胞比例0.005％；生化检查：总胆红素49.8 µmol/L、间接胆红素40.1 µmol/L、LDH 2 304 U/L；铁蛋白33 510 ng/ml，可溶性CD25 4 915 pg/ml；直接抗人球蛋白试验（direct antiglobulin test，DAT）示抗IgG及抗C3d均阳性（1：256）；补体活化指标C5b-9升高（566.7 ng/ml）；相关检查排除了血栓性血小板减少性紫癜、阵发性睡眠性血红蛋白尿症及系统性自身免疫病；外周血宏基因二代测序示巨细胞病毒（CMV）序列数48，CMV-DNA载量为1.8×10^3^拷贝数/ml。

2026年1月27日给予甲泼尼龙（80 mg/d）免疫抑制，伊普可泮（200 mg/次，每天2次）抑制补体旁路活化，并予抗感染、护胃、营养支持及间断输注洗涤红细胞。1月29日，患者突发精神异常，烦躁不安，此时HGB降至34 g/L，总胆红素35.0 µmol/L，考虑与重度溶血所致的中枢神经系统功能紊乱相关，予丙泊酚及右美托咪定镇静，并加用静脉丙种球蛋白（20 g/d×3 d）。1月31日，复查铁蛋白仍较高（10 370 ng/ml），网织红细胞比例降至0.001％，甲泼尼龙加量至120 mg/d。为明确贫血原因，排除噬血细胞综合征等可能，于2月2日行骨髓穿刺+活检。骨髓形态示骨髓增生活跃（+），粒/红系比明显偏高；病理及免疫分型未见淋巴瘤累及和相关噬血现象，支持AIHA诊断。患者网织红细胞比例极低，但骨髓红系未见明确受抑，提示可能存在免疫介导的继发性纯红细胞再生障碍样改变，而非原发性骨髓造血功能障碍。考虑患者为危重AIHA，激素及丙种球蛋白效果欠佳，于2月6日予利妥昔单抗600 mg每周1次治疗。

2月7日，患者突发一过性意识丧失伴血压下降，此时感染指标及体温正常，考虑血压下降主要与溶血加重有关（HGB降至26 g/L，尿色加深，总胆红素升至81.8 µmol/L）。经扩容、升压后恢复。同时，为阻断补体终末通路，予依库珠单抗900 mg。鉴于溶血危象加重，于2月8日及9日连续行血浆置换2次清除循环中游离抗体及炎性介质，并于2月9日予环磷酰胺600 mg。多线综合治疗后，患者尿色一度变浅，且铁蛋白、网织红细胞、LDH及游离血红蛋白等指标有所好转，但疗效不显著，且48 h内再次出现酱油色尿，HGB持续低水平波动（24～40 g/L），LDH仍较高。患者对前期治疗反应欠佳，且每次输注红细胞后均出现超急性溶血反应，提示患者体内可能存在大量已结合于红细胞表面的IgG型抗体及C3d补体成分，单纯血浆置换无法去除致敏红细胞，难以控制溶血。

经与输血科紧急会诊，决定实施WBE作为抢救性治疗。输血科团队在ABO血型及RhD抗原相合基础上，进一步完成Rh血型系统C、c、E、e抗原精准匹配。于2月10日实施WBE，建立体外循环通路，置换过程约171 min，估算患者总血容量为3 311 ml，置换出患者血浆1 810 ml及红细胞734 ml，并同步输注匹配的异体血浆及红细胞。患者全程生命体征平稳，无输血相关不良反应。术后24 h内患者尿色由浓茶色迅速转为淡黄色，且未再出现酱油色尿。

2月11日，总胆红素由术前87.6 µmol/L降至20.4 µmol/L，LDH由峰值2 304 U/L降至481 U/L（图1）。2月14日加用西罗莫司2 mg/d调节免疫治疗。2月15日HGB升至52 g/L，且后续输注红细胞未再出现溶血表现。2月19日，患者HGB稳定在60 g/L以上，尿色正常。但出现白细胞、血小板下降（WBC由术前8.52×10^9^/L降至3.90×10^9^/L，PLT由术前88×10^9^/L降至41×10^9^/L），考虑WBE后正常反应，予海曲泊帕及间断粒细胞集落刺激因子对症治疗，患者血象逐渐恢复至安全水平。

**图1:**
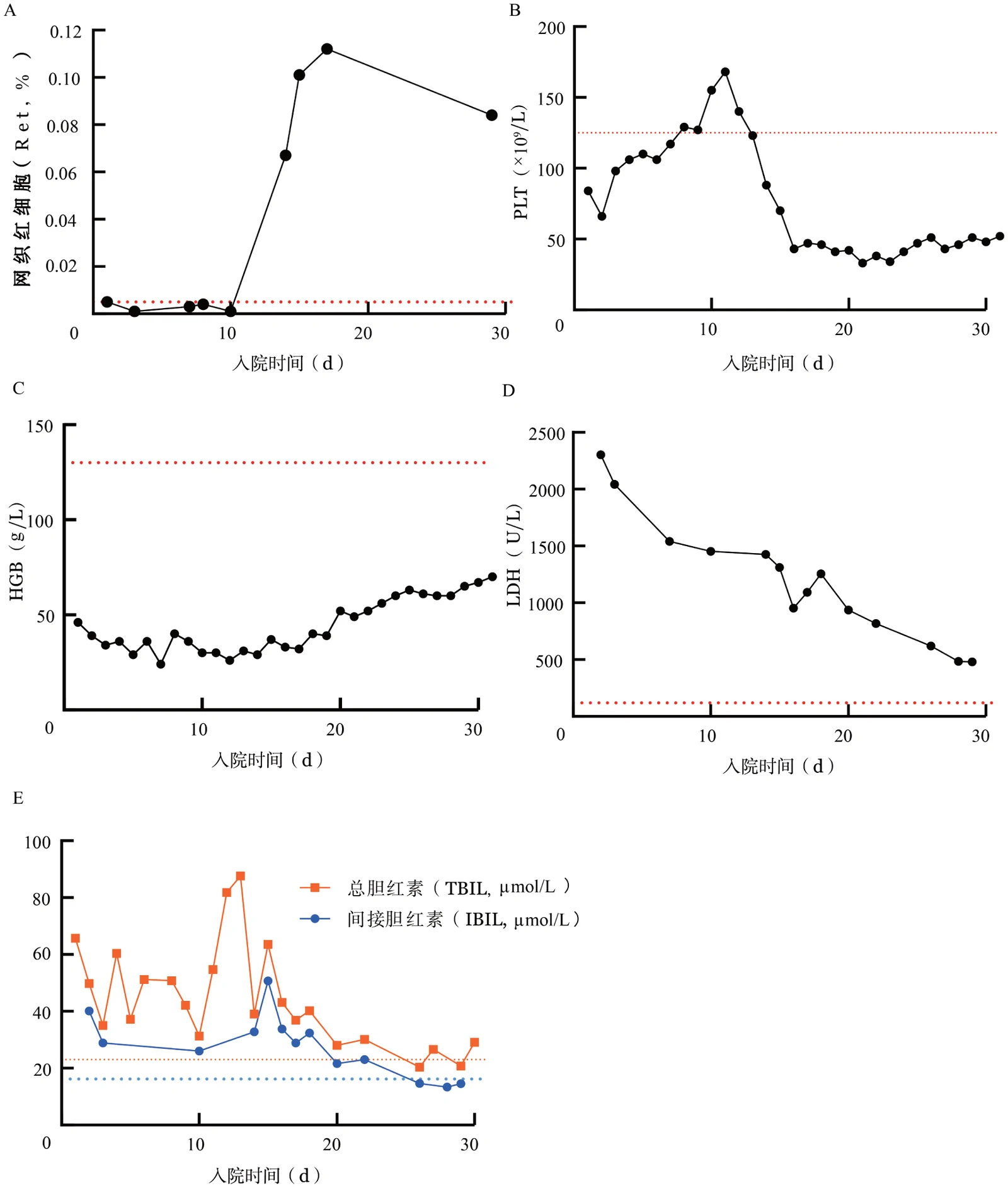
霍奇金淋巴瘤合并危重自身免疫性溶血性贫血患者入院后溶血相关指标及血小板动态趋势 **A** 入院后网织红细胞（Ret）变化情况；**B** 入院后PLT变化情况；**C** 入院后HGB变化情况；**D** 入院后LDH变化情况；**E** 入院后总胆红素和间接胆红素变化情况 **注** PLT：血小板计数；HGB：血红蛋白；LDH：乳酸脱氢酶；虚线为实验室正常参考值上限

2月22日，患者神志清楚，循环稳定，脱离输血及升压支持，转入血液科普通病房继续治疗。于3月3日完成第4次利妥昔单抗治疗，随访至2026年4月13日，患者HGB 108 g/L、PLT 109×10^9^/L，血象稳定，未见溶血复发。

## 讨论及文献复习

AIHA患者红细胞在短时间内被大量破坏，HGB急剧下降，常伴有酱油色尿、循环障碍及多器官功能损害，对糖皮质激素及免疫抑制剂等治疗无反应者，病死率极高[4]。本例患者因接受PD-1单抗治疗经典型HL，出现危重AIHA，DAT示抗IgG及抗C3d均强阳性，补体活化指标C5b-9显著升高。虽经糖皮质激素、静脉丙种球蛋白、利妥昔单抗、伊普可泮、依库珠单抗及血浆置换等多线治疗，溶血仍无改善，并出现精神异常、循环障碍，最终通过WBE成功阻断溶血，HGB由24 g/L升至70 g/L，尿色迅速转清，LDH由2 304 U/L降至481 U/L。本病例为难治性危重AIHA的挽救治疗提供了重要参考。

AIHA可作为淋巴瘤的副肿瘤综合征出现，约50％的AIHA患者由淋巴增殖性疾病或其他自身免疫性疾病引起[5]–[6]。HL继发AIHA相对罕见，但病情较重，可发生于淋巴瘤诊断前、诊断时、复发时或完全缓解后[7]。Pinczés等[8]报道563例HL中AIHA发生率为1.4％，且此类患者短期死亡率增加。Xing等[5]发现继发性AIHA患者较原发性患者年龄更大、缓解期更短、复发率和死亡率更高。HL继发AIHA患者对常规糖皮质激素治疗反应通常较差，这为本例的难治性提供了部分解释。

近年来，PD-1单抗等免疫检查点抑制剂（immune checkpoint inhibitor，ICI）广泛应用于淋巴瘤治疗，其通过阻断PD-1/PD-L1信号轴解除T细胞活化负向调控，但也可致外周免疫耐受失衡，诱发免疫相关不良事件（immune-related adverse event，irAE）[9]。Nishimura等[10]通过PD-1基因敲除小鼠模型首次证实PD-1缺失可导致自身反应性T细胞扩增及自身抗体产生。AIHA是ICI相关血液学irAE常见类型[11]，病死率约15％[12]。Tanios等[13]报道ICI相关AIHA的中位发病时间为10（2～78）周。多数PD-1抑制剂相关AIHA对糖皮质激素有效，部分联合丙种球蛋白或血浆置换后可控制病情[14]–[15]。

分析本例病因：PD-1抑制剂相关irAE是急性溶血危象的主要诱因，HL则为免疫稳态失衡提供了易感性基础。支持PD-1抑制剂为主要诱因的证据包括：①末次PD-1抑制剂用药至AIHA发病约38 d，符合文献报道的发病时间范围[13]；②患者既往有严重肺炎、皮疹等典型irAE表现；③DAT示抗C3d强阳性及C5b-9显著升高，符合补体介导的血管内溶血特征。HL的背景作用亦不可忽视：患者HL病情控制稳定，规律复查胸部CT，末次检查（2026年2月27日）示肺部病灶较前相仿，提示淋巴瘤在此期间未出现明显活动，且骨髓活检未见HL累及。综上，AIHA暴发时间与PD-1末次用药高度吻合，且有明确既往irAE史及补体激活证据，提示PD-1抑制剂为直接诱因，HL相关免疫紊乱则提供了易感背景，其机制可能为两种因素的叠加效应。

对于常规治疗无效的危重AIHA，WBE提供了一种新的挽救手段。WBE是在血浆置换基础上发展而来的技术，置换液由供体红细胞和新鲜血浆组成，可同时清除致敏红细胞、游离抗体及补体成分，并及时补充健康红细胞。关于WBE的禁忌证与适应证，既往文献缺乏统一明确的标准，结合文献检索及本中心经验，总结其适应证主要包括：危及生命的溶血[16]–[17]、新生儿高胆红素血症[18]、寄生虫高负荷[19]–[20]、部分中毒[21]。相对禁忌证包括对血制品严重过敏、血流动力学不稳[22]。此外，采用枸橼酸抗凝时，出血性卒中或缺血性卒中伴高出血转化风险者禁用[22]。操作方面，WBE是采用血细胞分离机及双针分离管路，建立外周静脉-静脉通路，每次置换总量一般为患者总血容量的50％～80％[23]。置换量计算原则：首先估算总血容量，置换血浆容量＝总血容量×（1−红细胞压积）×置换倍数，置换红细胞容量＝总血容量×红细胞压积×置换倍数[24]。

Pei等[25]通过苯肼诱导的药物性溶血性贫血（drug-induced hemolytic anemia，DIHA）大鼠模型证实WBE可降低死亡率、改善贫血指标，机制涉及抑制氧化应激及减轻炎症反应。Li等[26]对30例常规治疗无效的重症AIHA患者实施WBE，结果显示26例（86.7％）患者的HGB立即上升，总胆红素及抗体滴度下降。Jiang等[17]进一步证实WBE提升HGB效果优于传统治疗，且认为HGB<40 g/L是启动WBE的合适时机。本例患者在连续2次血浆置换无效后启用WBE，术后尿色即刻转清，溶血指标显著改善，HGB稳步回升，验证了WBE在危重难治性AIHA中的价值。本病例提供了在包括补体抑制剂、利妥昔单抗、血浆置换在内的多线治疗失败后，WBE作为挽救手段仍能快速阻断溶血的临床证据；为国内首次报道将WBE应用于PD-1抑制剂相关且合并HL复杂背景的极难治性、危重AIHA，并且提供了Rh系统抗原精细匹配的操作范例。WBE的成功实施高度依赖输血科团队在血制品充足保障、精准血型匹配及WBE操作中的深度参与，以及多学科团队的协同配合。

本研究为单病例报告，结论外推性有限，患者接受WBE联合多种药物治疗，难以精确评估单一疗法的贡献。此外，未系统开展T细胞亚群及功能检测，限制了机制探讨。WBE对设备及技术要求较高，其临床推广应用仍受到一定限制。由于随访时间有限，本病例的远期疗效及预后有待进一步观察。

## References

[1] Michel M, Crickx E, Fattizzo B (2024). Autoimmune haemolytic anaemias[J]. Nat Rev Dis Primers.

[2] Kuter DJ (2022). Warm autoimmune hemolytic anemia and the best treatment strategies[J]. Hematology Am Soc Hematol Educ Program.

[3] Lin DM, Becker J, Wu Y (2020). How do I perform whole blood exchange?[J]. Transfusion.

[4] Destival JB, Urbina T, Bougouin W (2025). Severe autoimmune hemolytic anemia in ICU: a place for emergency plasma exchange? A French multicenter retrospective study[J]. Crit Care.

[5] Xing L, Zhao M, Wang Y (2019). Characteristics of patients with autoimmune haemolytic anaemia secondary to lymphoproliferative disorder: A single-centre retrospective analysis[J]. Sci Rep.

[6] Barcellini W, Fattizzo B (2020). The changing landscape of autoimmune hemolytic anemia[J]. Front Immunol.

[7] Dimou M, Angelopoulou MK, Pangalis GA (2012). Autoimmune hemolytic anemia and autoimmune thrombocytopenia at diagnosis and during follow-up of Hodgkin lymphoma[J]. Leuk Lymphoma.

[8] Pinczés LI, Szabó R, Miltényi Z (2021). The impact of autoimmune cytopenias on the clinical course and survival of Hodgkin lymphoma[J]. Int J Hematol.

[9] Rogers BB, Zawislak C, Wong V (2021). Management of hematologic adverse events associated with immune checkpoint inhibitors[J]. J Adv Pract Oncol.

[10] Nishimura H, Nose M, Hiai H (1999). Development of lupus-like autoimmune diseases by disruption of the PD-1 gene encoding an ITIM motif-carrying immunoreceptor[J]. Immunity.

[11] Kroll MH, Rojas-Hernandez C, Yee C (2022). Hematologic complications of immune checkpoint inhibitors[J]. Blood.

[12] Davis EJ, Salem JE, Young A (2019). Hematologic complications of immune checkpoint inhibitors[J]. Oncologist.

[13] Tanios GE, Doley PB, Munker R (2019). Autoimmune hemolytic anemia associated with the use of immune checkpoint inhibitors for cancer: 68 cases from the Food and Drug Administration database and review[J]. Eur J Haematol.

[14] Zhang X, Gao BX, Guo CY (2023). A 71-year-old male with a life-threatening recurrence of hemolytic anemia, thrombocytopenia, and acute kidney injury after pembrolizumab therapy: a case report[J]. BMC Geriatr.

[15] Tao Y, Han J, Li Y (2022). Autoimmune hemolytic anemia in patients with relapsed Hodgkin's lymphoma after treatment with penpulimab, a monoclonal antibody against programmed death receptor-1[J]. Invest New Drugs.

[16] 国家皮肤与免疫疾病临床医学研究中心, 中国系统性红斑狼疮研究协作组, 中华医学会风湿病学分会 (2025). 中国系统性红斑狼疮诊疗指南(2025版)[J]. 中华医学杂志.

[17] Jiang Y, Zhao HJ, Luo H (2022). Efficacy of whole-blood exchange transfusion in refractory severe autoimmune haemolytic anaemia secondary to systemic lupus erythematosus: a real-world observational retrospective study[J]. Front Immunol.

[18] Kemper AR, Newman TB, Slaughter JL (2022). Clinical practice guideline revision: management of hyperbilirubinemia in the newborn infant 35 or more weeks of gestation[J]. Pediatrics.

[19] Lin J, Huang X, Qin G (2018). Manual exchange transfusion for severe imported falciparum malaria: a retrospective study[J]. Malar J.

[20] Krause PJ, Auwaerter PG, Bannuru RR (2021). Clinical practice guidelines by the infectious diseases society of America (IDSA): 2020 guideline on diagnosis and management of babesiosis[J]. Clin Infect Dis.

[21] Iolascon A, Bianchi P, Andolfo I (2021). Recommendations for diagnosis and treatment of methemoglobinemia[J]. Am J Hematol.

[22] Mekontso Dessap A, Dauger S, Khellaf M (2025). Guidelines for the management of emergencies and critical illness in pediatric and adult patients with sickle cell disease[J]. Ann Intensive Care.

[23] 治疗性单采过程管理与质量控制专家共识编写组 (2023). 治疗性单采过程管理与质量控制专家共识[J]. 临床输血与检验.

[24] Connelly-Smith L, Alquist CR, Aqui NA (2023). Guidelines on the use of therapeutic apheresis in clinical practice-evidence-based approach from the Writing Committee of the American Society for apheresis: the ninth special issue[J]. J Clin Apher.

[25] Pei S, Wang Y, Yao R (2025). Whole blood exchange ameliorates acute hemolytic anemia by reducing inflammation and oxidative stress in rats[J]. FASEB J.

[26] Li BJ, Yuan X, Jiang YJ (2015). Retrospective analysis of 30 severe autoimmune hemolytic anemia patients treated by whole blood exchange transfusion[J]. Transfusion.

